# Soluble and membrane-bound protein carrier mediate direct copper transport to the ethylene receptor family

**DOI:** 10.1038/s41598-019-47185-6

**Published:** 2019-07-24

**Authors:** Claudia Hoppen, Lena Müller, Sebastian Hänsch, Buket Uzun, Dalibor Milić, Andreas J. Meyer, Stefanie Weidtkamp-Peters, Georg Groth

**Affiliations:** 1 Institute of Biochemical Plant Physiology, Heinrich Heine University Düsseldorf, Universitätstraße 1, Düsseldorf, 40225 Germany; 2 Center for Advanced Imaging (CAi), Heinrich Heine University Düsseldorf, Universitätstraße 1, Düsseldorf, 40225 Germany; 3 Department of Structural and Computational Biology, Max Perutz Labs, Campus-Vienna-Biocenter 5, University of Vienna, 1030 Wien, Austria; 4 INRES – Chemical Signalling, University of Bonn, Friedrich-Ebert-Allee 144, 53113 Bonn, Germany

**Keywords:** Biophysical chemistry, Plant hormones, Biochemistry, Proteins

## Abstract

The plant hormone ethylene is a key regulator of plant growth, development and stress adaption. Ethylene perception and response are mediated by a family of integral membrane receptors (ETRs) localized at the ER-Golgi network. The biological function of these receptors relies on a protein-bound copper cofactor. Nonetheless, molecular processes and structures controlling assembly and integration of the metal into the functional plant hormone receptor are still unknown. Here, we have explored the molecular pathways of copper transfer from the plant cytosol to the ethylene receptor family by analyzing protein–protein interactions of receptors with soluble and membrane-bound plant copper carriers. Our results suggest that receptors primarily acquire their metal cofactor from copper transporter RESPONSIVE-TO-ANTAGONIST-1 (RAN1) which has been loaded with the transition metal beforehand by soluble copper carriers of the ATX1-family. In addition, we found evidence for a direct interaction of ETRs with soluble chaperones ANTIOXIDANT-1 (ATX1) and COPPER TRANSPORT PROTEIN (CCH) raising the possibility of a direct copper exchange between soluble chaperones and receptors.

## Introduction

Copper is an essential cofactor for many metalloproteins in all living organisms^1^. However, as the transition metal is toxic in the free monovalent form, copper homeostasis and delivery to the molecular targets in different cellular compartments is strictly controlled by specific protein carriers^2–4^. A main site for copper in plants controlling growth and development are the proteins of the ethylene receptor family^5,6^ which reside in the ER and Golgi membrane^7–10^. Naturally, this localization of the receptors requires the transport of monovalent copper from the plasma membrane via the cytoplasm to the apoproteins in the ER-Golgi membranes. The identity of the copper carriers involved in this task and their cellular routes are not yet completely known. Mutagenesis studies suggest that the metal cofactor is supplied by the P_1B_-type ATPase RAN1 (HMA7)^11,12^ as reduced RAN1 function was shown to alter ligand specificity of the ethylene receptor family^11–13^. Addition of copper to the growth medium of mutant plants restored these defects highlighting the importance of effective copper delivery to the receptors. The ability of RAN1 to mediate biogenesis of functional receptors^13^ favors direct interaction of the copper transfer ATPase with the receptors and suggests that both proteins are located - at least transiently - in the same subcellular compartment. However, to date neither their physical interaction nor the localization of RAN1 in the plant cell are known even though sequence homology with the mammalian Menkes/Wilson P-type copper ATPase suggests that RAN1 is localized at the Golgi membrane^14^. Other candidates for copper supply to the ethylene receptor family at the ER-Golgi network are the soluble copper chaperones of the ATX1 family (ATX1 and CCH) which showed direct interaction with RAN1 in yeast two-hybrid studies^15^. Several interactions between heavy metal transporting P-type ATPases (HMAs) and ATX1 chaperones have been reported for various species^16–18^ and recent studies also confirmed direct interaction between RAN1 and ATX1 in higher plants at the endomembrane system^15,19,20^.

In summary, the observations made so far lead to the hypothesis that copper is transferred from cytosolic ATX1 to RAN1 at the ER-Golgi and further transferred within these membranes to the ethylene receptor family. But in fact, the molecular processes and precise mechanism of copper transfer and copper assembly in the ethylene receptors are largely unknown and information on the biogenesis of the functional metallo-form of the receptors is sparse.

## Results and Discussion

**Figure 1:**
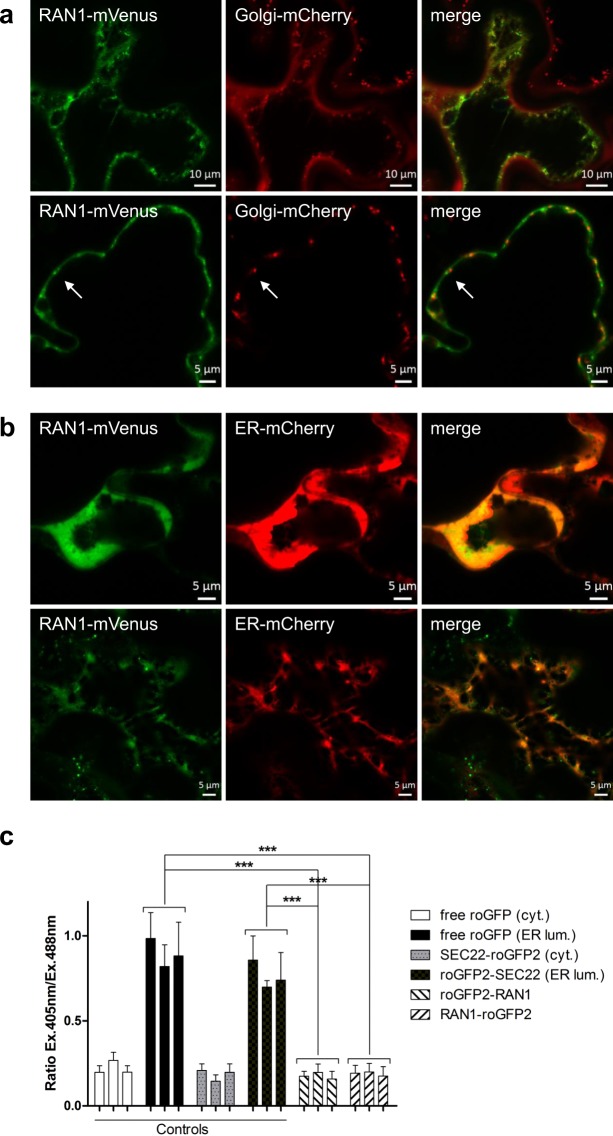
Subcellular Localization and Membrane Topology of RAN1 *in planta*. (**A)** Coexpression of RAN1-mVenus with a Golgi-mCherry marker show no clear colocalization of both proteins. Indicated by arrow are vesicle-like structures observed. Note, that these do not colocalize with the Golgi marker. (**B)** Colocalization of RAN1-mVenus with an ER-mCherry marker demonstrates that RAN1 is localized mainly at the ER membrane. (**C**) roGFP2 studies addressing the redox state of N- or C-terminally tagged fusions of RAN1 reveal that both termini are exposed to the cytosolic side of the ER membrane. Free roGFP2 targeted to either the ER or the cytosol together with N- or C-terminally tagged fusions of SEC22, a single membrane spanning protein, were used for internal control of RAN1 termini localization. Data shown represent three individual infiltrations per construct and ratios were calculated from 10 images per infiltration. Asterisks indicate significance level (p ≤ 0.0003) determined using Student’s t-test. Between RAN1-fusions and cytosolic control construct no significant difference could be detected (p > 0.5).

In this study, we analyzed the copper carriers ATX1, CCH and RAN1 for their interactions with the ethylene receptor ETR1 to explore the molecular pathways of copper transfer to the ethylene receptor family. Initially, we determined subcellular localization and topology of RAN1 in plant cells which had been inferred based on homology with other eukaryotic P-type HMAs, but have not been resolved experimentally yet^19^. Non-plant HMAs have been shown to shuttle between the plasma membrane and the *trans*-Golgi network depending on copper(I) availability as the mammalian Menkes and Wilson protein, or are presumed to act in the late or *post*-Golgi network as the yeast homologue ccc2^21,22^. To resolve this issue for RAN1 we applied an inducible system for transient expression in *N*. *benthamiana*^23^, allowing low expression levels of a fluorescently marked protein of interest in living cells. Notably, we identified clear colocalization of RAN1 with an ER marker (Fig. 1B) raising the possibility of physical interaction of the RAN1 copper transporter with the ER localized ethylene receptor family. Localization of RAN1 at the ER membrane was observed for different time points post induction excluding any impact of the expression level on localization (Fig. S1A). In contrast, no colocalization was found when RAN1-mVenus was coexpressed with a mCherry-tagged Golgi marker protein (Fig. 1A), although certain vesicle-like structures of RAN1-mVenus were detected at these conditions. However, these structures do not colocalize with the Golgi marker (Fig. 1A, lower panel). Finally, subcellular localization of soluble copper chaperones ATX1 and the plant specific homologue CCH were probed (Fig. S1). Both proteins show no colocalization neither with the ER- nor the Golgi marker. Instead, the diffuse appearance of the mVenus-tagged copper chaperones observed in confocal microscopy supports a cytosolic localization of these proteins.

Next, we studied the membrane topology of RAN1 at the ER membrane. HMAs share a similar overall structure and topology consisting of typically six or eight transmembrane helices, the membrane-external actuator domain (A domain) and the ATP-binding domain (ATP domain), and for most of them N- and occasionally C-terminal extensions containing ATX1-like metal-binding domains^24^. RAN1 is predicted to contain six to ten transmembrane domains (TMDs) and a long N-terminal extension. Different bioinformatic algorithms, however, predict different orientations of the N-terminus relative to the membrane (ARAMEMNON^25^) raising the question for the exact localization of the N-terminus. To further address this question, we made use of the redox-based topology assay (ReTa)^26^. In this assay, the redox-sensitive probe roGFP2 is fused to either the N- or the C-terminus of an ER-resident membrane protein of interest. After transient expression in tobacco leaves the fused probe self-indicates the orientation of the protein due to the steep redox gradient across the ER membrane with reducing conditions on the cytosolic face of the membrane and oxidizing conditions in the lumen. Studies of various RAN1-roGFP2 reporter constructs which are summarized in Fig. 1C revealed that both termini localize to the cytosolic side of the ER membrane indicating that the protein has an even number of TMDs.

**Figure 2:**
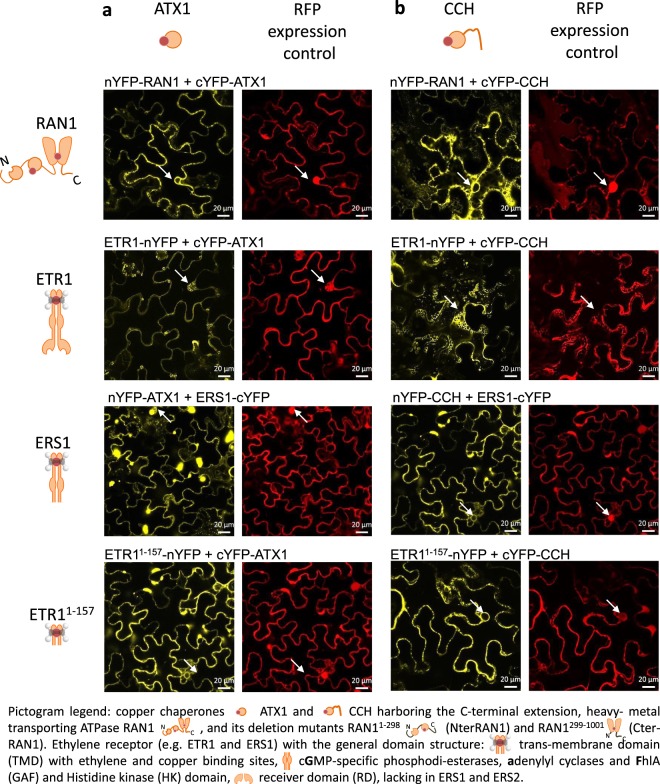
*In planta* Bimolecular Fluorescence Complementation (BiFC) studies on the interaction of RAN1 or type-I ethylene receptors ETR1/ERS1 with soluble copper chaperones ATX1 and CCH. BiFC studies reveal protein interaction of RAN1 and ATX1 (**A**). Fluorescence complementation was also detected for RAN1 and CCH (**B**) indicating that both soluble chaperones of the ATX1-family interact with RAN1 *in vivo*. BiFC studies on the interaction of soluble copper chaperones ATX1 and CCH with type-I ethylene receptor ETR1 and ERS1 indicate a direct protein interaction of receptors with soluble copper chaperones. RFP expression acts as an infiltration control for the BiFC vector and is constitutively expressed (d35S). Constructs containing full-length ETR1 were under control of an inducible promoter, whilst constructs containing ERS1 and ETR1^1–157^ were constitutively expressed (d35S). Arrows indicate the meshed like structure or nucleus envelope staining, typical for the ER, visible only for the complementation signal (YFP) but not for free RFP. nYFP and cYFP symbolize the YFP fragment fused either N- or C-terminal to the protein of interest.

To test for *in vivo* interaction of ethylene receptors, RAN1 and copper chaperones of the ATX1-family at the ER we used bimolecular fluorescence complementation (BiFC). First, we studied the interaction of RAN1 with soluble copper chaperones ATX1 and CCH in this set-up (Fig. 2A,B, upper panel, Fig. S2). Here, we used the RAN1/ATX1 BiFC pair as positive control in our set-up to demonstrate correct protein expression as interaction of both copper proteins has clearly been shown in previous studies^15,19^. Notably, the ATX1-like chaperone CCH which differs from all known ATX1 homologues by a C-terminal extension (~45 amino acids), showed fluorescence complementation, similar to RAN1 and ATX1 in our experiments. Hence, based on these data we conclude that both soluble copper chaperones of the ATX1-family present in higher plants are able to interact with RAN1 although previous yeast-two-hybrid (Y2H) studies failed to demonstrate physical interaction of CCH with the plasma membrane localized P-type HMA *At*HMA5 or with RAN1, respectively^15,27^. Receptor-like protein CLAVATA2 (CLV2) localizing to the ER and glutathione-S-transferase (GST) localizing in the cytosol were used as negative controls to ensure that the observed YFP emission result from fluorescence complementation of a specific interaction, but not from nonspecific self-assembly induced by the overexpression of the candidate interactors. No detectable YFP fluorescence emission was observed when RAN1, ATX1 or CCH (Fig. S3) were tested with the CLV2 or GST control verifying the specificity and biological relevance of the observed RAN1-ATX1 and RAN1-CCH interactions.

**Figure 3:**
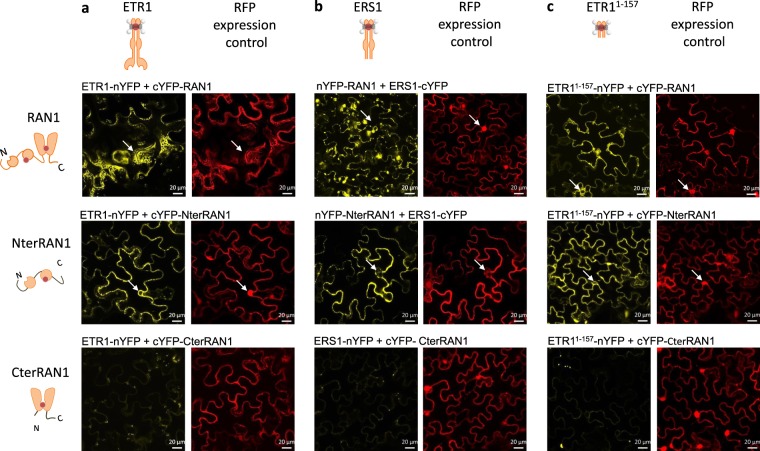
*In planta* Bimolecular Fluorescence Complementation (BiFC) studies on the interaction of RAN1 with type-I ethylene receptors ETR1 and ERS1. BiFC studies on RAN1 and deletion mutants reveal an *in vivo* interaction of RAN1 with receptors ETR1 (**A**) and ERS1 (**B**). Complementation is observed for full-length RAN1 and NterRAN1 but not for CterRAN1 indicating that the large N-terminal region of RAN1 mediates the interaction. Additionally, deletion mutant ETR1^1–157^ (**C**) was tested which also interacts with RAN1 and NterRAN1 demonstrating that the transmembrane part of the receptor seems to be sufficient for interaction. RFP expression acts as an infiltration control for the BiFC vector and is constitutively expressed (d35S). Constructs containing full-length ETR1 were under control of an inducible promoter whilst constructs containing ERS1 and ETR1^1–157^ were constitutively expressed (d35S). Arrows indicate the meshed like structure or nucleus envelope staining, typical for the ER, visible only for the complementation signal (YFP) but not for free RFP. nYFP and cYFP symbolize the YFP fragment fused either N- or C-terminal to the protein of interest.

To further clarify copper transfer on the receptors, fluorescence complementation of RAN1 and type-I ethylene receptors ETR1 and ERS1 was analyzed (Figs 3, S2). Indeed, fluorescence complementation was detected with both receptor isoforms indicating physical interaction with RAN1 at the ER membrane. Direct interaction of RAN1 and receptors has not been considered an essential element of copper transfer yet as previous studies have shown that yeast copper transporter Ccc2 is able to restore ethylene-binding affinity in genetically engineered *Saccharomyces cerevisiae* expressing ETR1^13^. Based on the results of our topology studies we tested different truncations of RAN1 to pinpoint the domains interacting in the receptors and the copper transporter. The mutant consisting of the large cytosolic N-terminal region only was named NterRAN1 (amino acids 1–298), whereas the construct lacking this ATX1-like domain was termed CterRAN1 (amino acids 299–1001). Additionally, a construct consisting only of the transmembrane sensor domain with the putative copper(I) binding site of ETR1, ETR1^1–157^, was tested. Complementation with ETR1, ERS1 or truncated ETR1^1–157^ was observed only with full-length RAN1 and the NterRAN1 domain, but not with the CterRAN1 truncation indicating that the N-terminal region is directly involved in the interaction. Hence, copper transfer by RAN1 on the receptors is probably catalyzed by the membrane-external ATX1-like domain of the metal transporter. In addition complementation with ETR1^1–157^ indicates that the transmembrane part is sufficient to mediate interaction. As for the BiFC studies with RAN1 and soluble chaperones of the ATX1 family, ETR1, ETR1^1–157^ or ERS1 showed no YFP emission due to nonspecific self-assembly of the fluorophore as demonstrated in studies with the CLV2 or GST negative control (Fig. S3). Additionally, the subcellular localization of ETR1 truncated version ETR1^1–157^ was checked by coexpression with ER- and Golgi marker proteins. ETR1^1–157^ predominantly remains localized at ER membrane (Fig. S4).

We next asked whether soluble chaperones of the ATX1 family can directly interact with the ethylene receptor family. Remarkably, fluorescence complementation was detected for type-I receptors ETR1 and ERS1 with both soluble copper chaperones, ATX1 and CCH (Fig. 2A,B lower panels, Fig. S2). These results point on a new - so far unknown - route for intracellular copper transfer on the receptors. To confirm this interaction and to resolve the domains in the interacting proteins in further experiments we used the ETR1^1–157^ truncation. Also in this case, fluorescence complementation was observed indicating that the transmembrane part of the receptor is sufficient for the interaction with the soluble copper chaperones of the ATX1 family (for further evidence on the contribution of the transmembrane part see *in vitro* interaction studies). Taken together these studies suggest that soluble ATX1-like chaperons are able to transfer their copper load from the cytoplasm to RAN1 at the ER membrane which subsequently transfers the metal ion to the receptors at the same membrane. However, soluble chaperones may also bypass RAN1 and directly interact with the ethylene receptors at the ER membrane. Copper binding at ETR1 independent of RAN1 has already been shown in previous studies in genetically engineered yeast^13^. Mutants expressing ETR1 but lacking the RAN1 homolog Ccc2p show no ethylene binding activity. However, addition of copper sulfate at 300 µM concentration was able to restore ethylene binding activity in these mutants indicating that other copper carrier may provide the metal for the receptors as monovalent copper due to its cytotoxicity cannot exist in its free form in living organism.

**Figure 4:**
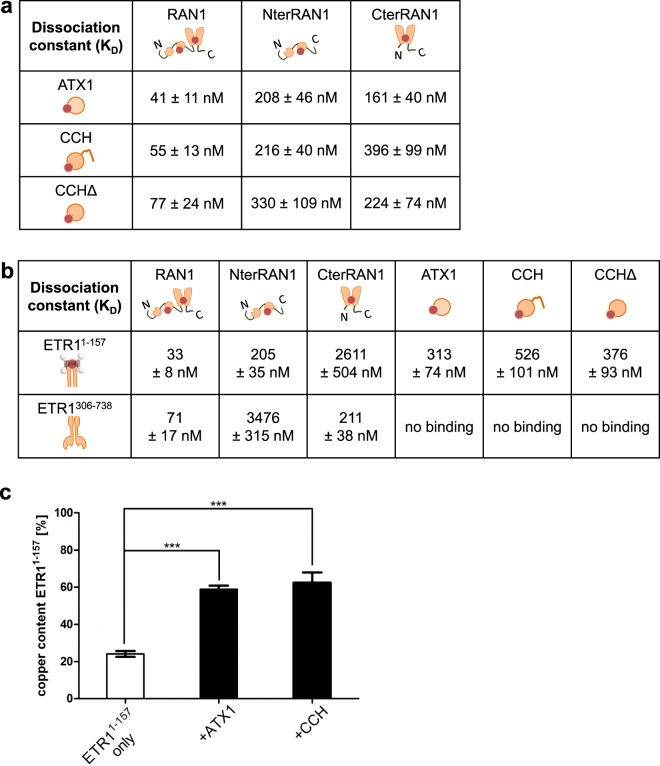
*In vitro* binding studies on RAN1, ETR1 and soluble copper chaperones by microscale thermophoresis (MST) (**A)** Dissociation constants of RAN1 and RAN1 truncation mutants with soluble copper chaperones ATX1, CCH and CCHΔ, respectively. (**B**) Dissociation constants of ETR1^1–157^ and ETR1^306–738^ with copper carriers RAN1, NterRAN1, CterRAN1, ATX1, CCH or CCHΔ, respectively. ETR1 interacts with both, the ATX-like chaperones and RAN1. Noteworthy, affinity of ETR1 for soluble copper chaperones is 10 fold lower than observed for ETR1 with full-length RAN1. For detailed binding curves see Fig. S3. (**C**) Copper transfer assay with purified recombinant Cu(I)-ATX1 or Cu(I)-CCH and ETR1^1–157^. Copper content of ETR1^1–157^ before (ETR1^1–157^ only) and after (+ATX1, +CCH) incubation with preloaded donor copper chaperone. Increase in copper loading after chaperone incubation indicates chaperone mediated copper transfer to ETR1. Asterisks indicate significance level (p ≤ 0.001) determined using Student’s t-test.

To further explore the copper transfer network to the receptors, binding affinities of purified copper proteins were determined by microscale thermophoresis (MST). In addition to ATX1, RAN1 and receptor ETR1 a deletion mutant of the soluble copper chaperone CCH, CCHΔ, lacking the C-terminal extension was purified and used for protein interaction studies. All proteins were cloned from *Arabidopsis*, expressed in *E*. *coli* and purified to homogeneity from the bacterial host (Fig. S6A). Functional folding and activity of recombinant proteins were verified by copper(I) binding and/or ATPase activity assays (Fig. S6B,C). *In vitro* binding studies of RAN1 with ATX1 or CCH revealed similar affinities (Figs 4A, S5A) of the copper transporter for both soluble chaperones with apparent dissociation constant of 41 ± 11 nM (ATX1) and 55 ± 13 nM (CCH). These data are in accordance to our previous BiFC analysis emphasizing that both types of chaperones are able to interact with RAN1 at similar affinities. Further binding studies using the CCHΔ deletion mutant show that the C-terminal region is not crucial for the interaction with RAN1 as indicated by a dissociation constant (K_D_ = 77 ± 24 nM) similar to full-length CCH. Additional studies on RAN1 deletion mutants with purified ATX1, CCH and CCHΔ show that the purified chaperons of the ATX1 family interact with both, NterRAN1 and CterRAN1, with similar dissociation constants ranging from 160 to 396 nM (Figs 4A, S5A). Consequently, besides transfer at the N-terminal ATX1-like domain direct copper transfer to the transmembrane transport site in RAN1 seems possible as reported for the *Archaeoglobus fulgidus* copper chaperone CopZ and ATPase CopA^28^.

To substantiate our BiFC studies indicating a direct interaction of the copper transport ATPase RAN1 and ethylene receptors at the ER membrane, MST measurements were performed on purified recombinant proteins which confirmed a specific, high affinity interaction of ETR1^1–157^ with full-length RAN1 (K_D_ = 33 ± 8 nM, Figs 4B, S5C). To further identify the RAN1 domain interacting with the transmembrane copper binding domain in the receptor, truncation mutants NterRAN1 and CterRAN1 were analyzed. In these studies, a dissociation constant of 205 ± 35 nM was obtained for the interaction of ETR1^1–157^ with NterRAN1. In contrast, a 10 fold higher K_D_ of 2611 ± 504 nM was found when ETR1^1–157^ was tested with the CterRAN1 mutant representing the transmembrane copper transport site of the HMA. Together, these data suggest that the interaction between RAN1 and ETR1 is mediated mainly by the N-terminal ATX1-like domain of RAN1. To test whether the soluble extra-membranous part of ETR1 also contributes to the interaction of both proteins, we initiated binding studies of full-length RAN1, CterRAN1 and NterRAN1 with ETR1^306–738^ (Figs 4 and S5). In these studies, we observed clear interaction of full-length RAN1 and ETR1^306–738^ with an affinity constant of 71 ± 17 nM suggesting that the extra-membranous part of ETR1 provides additional interaction sites and stabilizes the ETR1-RAN1 complex. In contrast to studies with ETR1^1–157^, NterRAN1 showed only weak affinity for ETR1^306–738^ (K_D_ = 3476 ± 315 nM) while CterRAN1 still provides high affinity interaction to ETR1^306–738^ (K_D_ = 211 ± 38 nM). Based upon these studies, we conclude that the RAN1–ETR1 interaction is mediated by both, binding of the N-terminal part of RAN1 to the transmembrane region of ETR1 and binding of the extra-membranous part of ETR1 to the C-terminal region of RAN1.

To further elaborate the role of the soluble metal carrier in copper transport to the receptors, binding studies were performed with purified ETR1 and chaperones ATX1, CCH or CCHΔ (Figs 4B, S3B), respectively. In these studies, ETR1 shows similar affinities for ATX1 and CCHΔ with apparent dissociation constants of 313 ± 74 nM (ATX1) and 376 ± 93 nM (CCHΔ). In contrast, the CCH wildtype has a lower binding affinity on ETR1 (K_D_ = 526 ± 101 nM) indicating that the C-terminal extension partially shields the interaction site. Nonetheless, all three soluble copper chaperones show clear interaction with ethylene receptor ETR1 *in vivo* and *in vitro* supporting copper exchange from the cytosolic copper chaperones to the ethylene receptors at the ER. No binding of soluble copper chaperones was observed with the extra-membranous part of the receptor (ETR1^306–738^) indicating that the interaction of receptors and soluble chaperones is entirely mediated by the transmembrane part, i.e. this part of the receptor is necessary and sufficient for the interaction (Figs 4 and S5). With that in mind, the observed approximately 10 fold higher affinity of ATX1-like chaperones for ETR1 compared to RAN1 (see respective K_D_^’^s in Figs 4 and S5) may result from the additional binding of RAN1 with the extra-membranous part of the receptor.

The studies presented so far provide compelling evidence for the interaction of soluble and membrane-bound copper carrier and ETRs. Based on the biological function of the copper carrier these interactions imply that the transition metal is transferred from the copper carrier to the cofactor binding site at the receptor. To demonstrate such chaperone-mediated copper transfer, soluble copper chaperones ATX1 and CCH were preloaded with the transition metal. The copper-loaded chaperones Cu(I)-ATX1 and Cu(I)-CCH then served as donors of the transition metal and were incubated with purified ETR1. Copper loading of receptors analyzed prior and after chaperone interaction shows clear differences (Fig. 4C) and reveals that the transition metal can be transferred between soluble chaperones of the ATX1 family and ETR1 in the receptor–chaperone complex.

**Figure 5:**
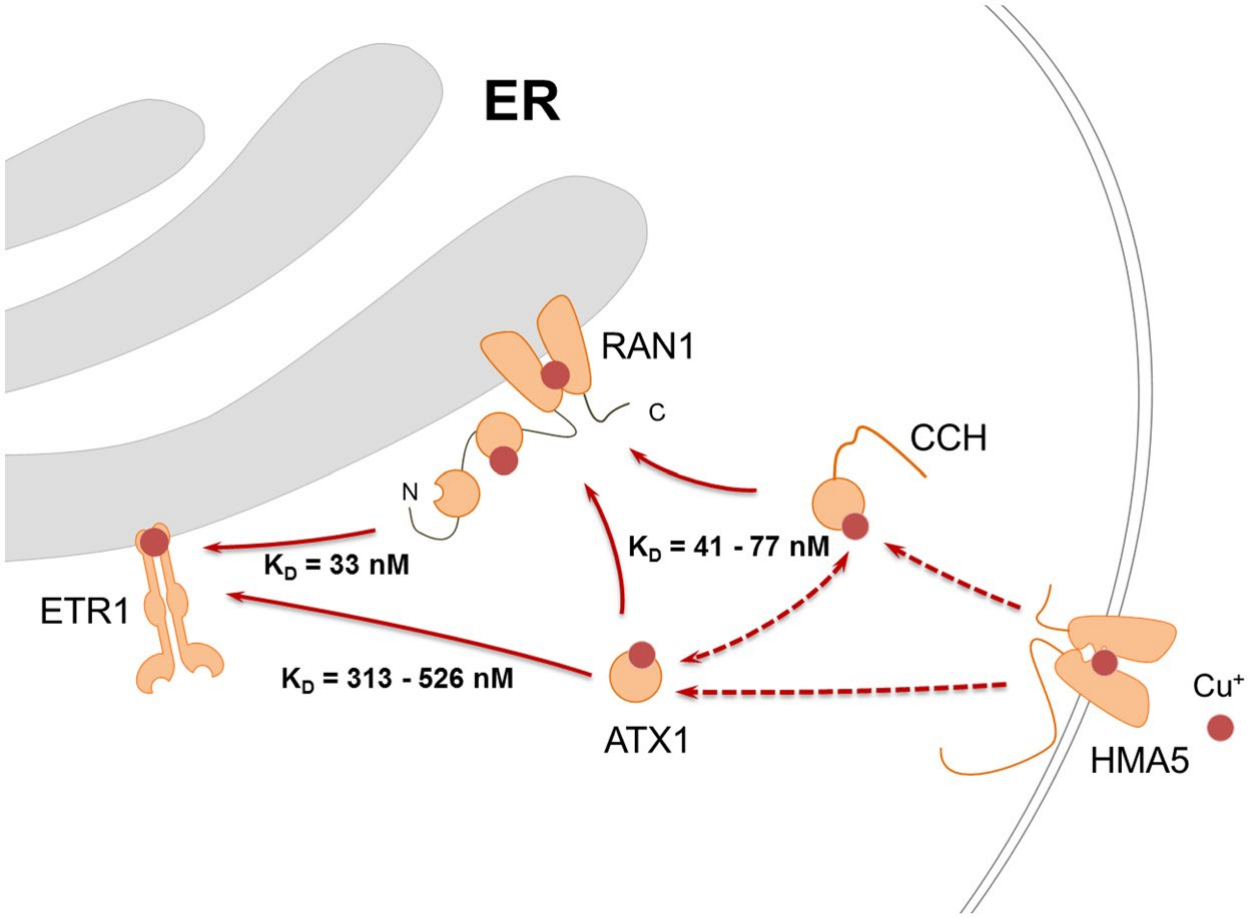
Proposed copper transport pathway of the transition metal to the ethylene receptor. Copper(I) is imported into the cell by HMA5. Two possible routes then deliver the monovalent cation to the ER localized receptors. The first route requires stepwise transfer of copper ions from soluble chaperones ATX1 or CCH to RAN1 followed by a transfer of the transition metal from RAN1 onto ETR1. The low dissociation constants determined for the related protein interactions suggest that this pathway is the major route. However, based on their *in vivo* and *in vitro* interaction a second alternative route of direct transfer of the metal cofactor from the soluble copper chaperones ATX1 or CCH to the receptors is also existing. Solid arrows correspond to interactions analyzed in this study; dashed arrows indicate interactions presumed from previous publications.

Our *in vivo* and *in vitro* studies indicate that receptors may acquire their essential copper cofactor on two routes, a RAN1-dependent pathway and a RAN1-independent pathway (Fig. 5). Observed binding affinities imply that the gradual transfer from ATX1 to RAN1 (K_D_ = 41 nM ± 11 nM) further to ETR1 (K_D_ = 33 nM ± 8 nM) is the primarily route. Binding studies on truncation mutants stress that the N-terminal ATX1-like domain of RAN1 plays a central role in this process. Noteworthy, the direct transfer of the metal ion from soluble ATX1-like chaperones in the cytosol (K_D_ ≥ 313 ± 74 nM) may represent an alternative copper route to the receptors. This route is supported by *in vivo* BiFC data and *in vitro* MST measurements. The 10 fold higher dissociation constant detected for the interaction of chaperones and receptors may indicate that this route is less favored and active only at certain physiological or developmental conditions. In this context, the expression pattern of ATX1 and RAN1 in *A. thaliana* shoots support a complementary role of both proteins at high and low copper level (i.e. in the presence of copper chelator BCS)^29^. At high copper level when RAN1 expression is downregulated^29^, direct interaction with ATX1 may ensure copper supply to the ethylene receptor family. Intriguingly, transcript levels of CCH are regulated converse to the expression of ATX1 but similar to RAN1 indicating a coherent regulation for RAN1 and CCH and the necessity for an “backup” copper route to the receptors^29^.

## Summary

Copper delivery in plants needs to be tightly controlled to avoid toxic effects of the highly reactive transition metal. Nevertheless, several proteins have been described that require copper in its monovalent form for functionality. In addition to plastocyanin and superoxide dismutase, ER/Golgi localized ethylene receptors are a major group of plant copper proteins. In summary, our studies evince the full path from the cytosolic ATX1 chaperone family to RAN1 and the ethylene receptors at the ER membrane. Moreover, they pinpoint the protein domains interacting in this copper cascade and for the first time indicate direct copper exchange between the cytosolic chaperones of the ATX1-family and the ethylene receptor family. However, further studies are necessary to resolve molecular structures and dynamics of the copper routes to the receptors and to substantiate the biological relevance of the ATX1/CCH–ETR interactions.

## Methods

### Cloning

Entry vectors for Gateway cloning were generated using the pENTR/D-TOPO Cloning Kit (Invitrogen) or via BP reaction of PCR products and pDONR221 vectors. Vectors for expression in *N*. *benthamiana* were generated by LR reaction of entry vectors (TOPO or pDONR221) and desired destination vectors (pAB^30^ or pBiFC-2in1^31^). For expression of full-length ETR1, an inducible BiFC vector, pBiFC-ind-CN, was cloned by combining features of pAB and pBiFC-2in1. For subcellular localization studies an inducible expression system was used^23^. BiFC^31^ and roGFP2^26^ studies were performed using d35-driven constitutive expression of the gene of interest. Constructs for heterologous expression of full-length and deletion mutants in *E*. *coli* were integrated by Gibson assembly^32^ into pETEV16b.

### Agroinfiltration and expression in *N*. *benthamiana*

Transformation and infiltration were performed as previously described^33^. Confocal laser scanning microscopy was performed using a Zeiss LSM780 laser scanning microscope in cooperation with the *Center for advanced imaging* (Cai) at Heinrich-Heine University Düsseldorf. mVenus fluorescence was excited at 488 nm (Laser intensity: 1.8–6%, emission: 503–556 nm), while mCherry fluorescence was excited at 561 nm (Laser intensity: 2.2–4%, emission: 570–624 nm). Pinhole was always adjusted to 1 Airy unit (AU). In addition, the chloroplast autofluorescence was detected in a separate detector between 625 nm and 735 nm in the same track as the mCherry signal.

### Ratiometric analyses of RAN1 topology

Gateway destination vectors, control constructs for roGFP, cloning of fusion constructs and agroinfiltration of 4-week-old *N*. *benthamiana* plants were performed as described in^26^. Ratiometric confocal imaging was done on a Zeiss LSM780 laser scanning microscope. roGFP fluorescence was excited at 405 nm and 488 nm. In total 30 images for each construct were collected (3 × 10, individual infiltrations) and used for ratio calculation. Image analyses was performed using ImageJ (32 bit v1.51t.) and ratiometric data were obtained by dividing images collected upon excitation at 405 nm by images collected by excitation upon 488 nm. Only pixels with intensity levels of at least 4 times higher than the background were taken into account and saturated pixels were excluded.

### Bimolecular fluorescence complementation (BiFC)

Gateway destinations vectors for the 2in1 cloning system and BiFC analyses were a kind gift from Dr. Christopher Grefen (Ruhr-Universität Bochum). Imaging of BiFC constructs was performed on a Zeiss LSM780 laser scanning microscope. YFP fluorescence was excited at 488 nm (Laser Intensity: 4.2–7%, Emission: 503–556 nm), while RFP fluorescence (infiltration marker) was excited at 561 nm (Laser Intensity: 2%, Emission: 570–624 nm). Pinhole was always adjusted to 1 Airy unit (AU). In addition, the chloroplast autofluorescence was detected in a separate detector between 625 nm and 735 nm in the same track as the mCherry signal.

### Heterologous expression in *E*. *coli*

For expression of *A. thaliana* ATX1, CCH, CCHΔ and NterRAN1, *E*. *coli* BL21 (DE3) or C43 (DE3) were transformed with pETEV16b-ATX1/CCH/CCHΔ/NterRAN1 or pETEV16b-RAN1/CterRAN1 and grown on 2YT-Agar plates (+Amp^120^) overnight at 37 °C. 100 ml 2YT medium (+Amp^120^) were inoculated and incubated overnight at 37 °C and 180 rpm. 5 ml were used to inoculate 500 ml 2YT medium (+Amp^120^). Cultures were grown to an OD_600_ of 0.5 and then cooled down to 16 °C. Expression was induced with 0.5 mM of IPTG when OD_600_ reached 0.8. Cultures were incubated overnight at 16 °C and 180 rpm. After harvest, cells were shock frozen in liquid nitrogen and stored at −20 °C. Expression and purification of AtETR1^1–157^ was performed as described previously for tomato ethylene receptors LeETR4 and NR^34^. Cells expressing RAN1 and CterRAN1 were stored at −80 °C. ETR1^306–738^ was expressed in *E*. *coli* and cells were stored as described in^35^.

### Purification of recombinant proteins

For purification of ATX1, CCH, CCHΔ, RAN1, NterRAN1, and CterRAN1 cells were resuspended in 5 ml/g of buffer A (50 mM HEPES, 300 mM NaCl, 10% (w/v) glycerol, 1 mM DTT, 0.002% (w/v) PMSF, DNaseI, pH 7.0-8.2). Cells were lysed using a cell disruption system (Constant Systems Limited) and centrifuged for 1 h at 100 000 × g for soluble proteins or 40 000 × g for membrane collection. Membrane proteins were solubilized by adding 1% (w/v) DDM and centrifuged at 230.000 × g after 1 h of gentle agitation. In both cases supernatants were loaded on a 5 ml HisTrap HP column, equilibrated with buffer A or buffer A + 0.1% (w/v) DDM using an ÄKTAPrime plus system (GE Healthcare). After a washing step with buffer A + 100 mM Imidazole, proteins were eluted with buffer B (buffer A + 500 mM imidazole). Elution fractions were pooled, concentrated by ultracentrifugation using Amicon Ultra-15 tubes (Merck, Darmstadt) and buffer exchanged using PD 10 desalting columns (GE Healthcare) equilibrated with buffer A. The protein solutions were aliquoted to 500 µl, shock frozen in liquid nitrogen and stored at −80 °C. Aliquots were thawed on ice and digested with 100 µg TEV protease for 1 h at room temperature or overnight at 4 °C to cleave off the 10xHis-tag. 500 µl were then loaded on a Superdex200 10/300 GL size exclusion column, equilibrated with buffer SEC (50 mM HEPES, 300 mM NaCl, pH 7–8.2) or buffer SEC + 0.1% (w/v) DDM. If necessary, elution fractions were concentrated. Successful purification, functionality and activity of the recombinant proteins were checked by SDS-PAGE, copper(I) binding ability and/or ATP hydrolysis (Fig. S6). ETR1^306–738^ was purified and stored as described in^35^.

### Labeling of purified proteins

For interaction studies by microscale thermophoresis, recombinant proteins were labeled with AlexaFluor488-NHS (Life Technologie) prior the final purification step by size exclusion. To this, recombinant protein was incubated with 2.5 fold amount of AlexaFluor488-NHS for 1 h at room temperature followed by digestion with TEV protease. 500 µl of labeled protein solution was loaded on a Superdex200 10/300 GL size exclusion column to separate aggregates, TEV protease and free dye from the protein of interest in a single step. Elution fractions were pooled and directly used in MST measurements.

### Microscale thermophoresis

Binding studies of purified recombinant proteins were performed in triplicates using a Monolith NT 115 and premium capillaries (NanoTemper Technologies) with 60% MST power and 60–70% LED power. In these studies RAN1 and CterRAN1 were labeled as described above and diluted to a concentration of 15 nM in buffer SEC-R (50 mM HEPES, 300 mM NaCl, 0.1% (w/v) DDM pH 7.5) and NterRAN1 was diluted to a concentration of 50 nM in buffer SEC (50 mM HEPES, 300 mM NaCl, pH 7.5) containing 0.05% (w/v) Tween20. Dilution series of ATX1, CCH and CCHΔ from 30 µM to 0.9 nM were prepared in buffer SEC (50 mM HEPES, 300 mM NaCl, pH 7.5). For measurements ETR1^1–157^, RAN1 and CterRAN1 were labeled as described above and diluted to a concentration of 20 nM in buffer SEC-R (50 mM HEPES, 300 mM NaCl, 0.1% (w/v) DDM pH 7.5). NterRAN1 was diluted to a concentration of 40 nM in buffer SEC (50 mM HEPES, 300 mM NaCl, pH 7.5) containing 0.05% (w/v) Tween20. Dilution series of ETR1^1–157^ from 10 µM to 0.3 nM was prepared in buffer SEC (50 mM HEPES, 300 mM NaCl, pH 7.5) with 0.015% (w/v) FosCholine-16. For measurements of ETR1^1-157^ and ATX1-like chaperones (ATX1, CCH and CCHΔ), soluble chaperones were labeled as described above and diluted to a concentration of 75 nM, 100 nM and 50 nM, respectively, in buffer SEC (50 mM HEPES, 300 mM NaCl, pH 7.5) with 0.015% FosCholine-16, and 0.05% Tween20. A serial dilution of ETR1^1–157^ from 7.5 µM to 0.2 nM was prepared in buffer SEC (50 mM HEPES, 300 mM NaCl, pH 7.5) containing 0.015% (w/v) FosCholine-16 and 0.05% (w/v) Tween20. For measurements with ETR1^306–738^ a serial dilution from 37 µM to 1.1 nM was prepared in buffer D (50 mM Tris, 250 mM NaCl, 0.015% (w/v) FosCholine16, 2.5 mM DTT, 20% (w/v) glycerol, cOmplete EDTA-free protease inhibitor cocktail, pH 8.5). Labeled RAN1, CterRAN1, NterRAN1, ATX1, CCH and CCHΔ were diluted to a final concentration of 50 nM in buffer D.

### His-Tag cleavage and metal loading of copper chaperones

Purified recombinant chaperones CCH and ATX1 were incubated over night with TEV protease in a 1:70 ratio at 4 °C to remove the 10x histidine tag. Processed chaperones were loaded with monovalent copper by incubation with the chromophoric BCA_2_-Cu(I)-chelate complex for 5 min (see also Fig. S6) until solution stays purple. Separation of copper-loaded, His-tag free chaperones was performed by size exclusion chromatography using a Superdex200 10/300 GL column operated on an ÄKTAPrime plus (both GE Healthcare). Chaperones were concentrated to 400 µM by ultrafiltration.

### Copper transfer assays from chaperones to receptor

To assess copper transfer from chaperones (ATX1 and CCH) to receptor (ETR1), 250 µL of purified histidine-tagged ethylene receptor ETR1^1–157^ with a concentration of 200 µM was bound to 150 mg Protino Ni-TED resin (Macherey-Nagel) equilibrated in 250 µL buffer X (50 mM Tris/HCl pH 8, 200 mM NaCl, 0.015% FosCholine-16) for 1 h at RT. Then, 250 µL of 400 µM purified fully copper-loaded chaperone was added and incubated together with resin-bound ETR1^1–157^ for 2 h at RT. Ni-TED material was spun down for 60 s at 14.000 rpm. The supernatant was collected and concentrated for further analysis. Steps were repeated for 30 min each at RT with 500 µL buffer X containing 100 mM, 250 mM and 500 mM imidazole, respectively. Fractions were analyzed by western blotting and SDS-PAGE. Protein concentration was calculated based on the absorption at 280 nm. Copper concentration in fractions exclusively containing ETR1^1–157^ was determined after chemical and thermal denaturation of the samples using 20% SDS and heating to 95 °C for 15 min. The copper released was re-complexed with 20 mM BCA and the amount of the chromophoric BCA_2_-Cu(I) chelate complex formed in this assay detected spectrophotometrically at 562 nm. Percentage of copper loading was calculated from protein and copper concentrations determined.

## Supplementary information

Supplemental Information
