# Supplementary material for: Soluble and membrane-bound protein carrier mediate direct copper transport to the ethylene receptor family
Source: Sci Rep. 2019 Jul 24;9:10715. doi: 10.1038/s41598-019-47185-6 (PMC6656775; doi:10.1038/s41598-019-47185-6)
Supplement: Supplementary file 1 — Supplemental Information [file 41598_2019_47185_MOESM1_ESM.docx]

**Soluble and membrane-bound protein carrier mediate direct copper transport to the ethylene receptor family**

Claudia Hoppen^1^, Lena Müller^1^, Sebastian Hänsch^2^, Buket Uzun^1^, Dalibor Milić^3^, Andreas J. Meyer^4^, Stefanie Weidtkamp-Peters^2^, Georg Groth^1,*^

^1^Institute of Biochemical Plant Physiology, Heinrich Heine University Düsseldorf, Universitätsstraße 1, Düsseldorf 40225, Germany

^2^Center for Advanced Imaging (CAi), Heinrich Heine University Düsseldorf, Universitätsstraße 1, Düsseldorf 40225, Germany

^3^Department of Structural and Computational Biology, Max Perutz Labs, University of Vienna, 1030 Wien, Austria

^4^INRES – Chemical Signalling, University of Bonn, Friedrich-Ebert-Allee 144, 53113 Bonn, Germany

*Correspondence should be addressed to G.G., e-mail: [georg.groth@hhu.de](mailto:georg.groth@hhu.de)

Supplemental Information (SI)

Figure S1


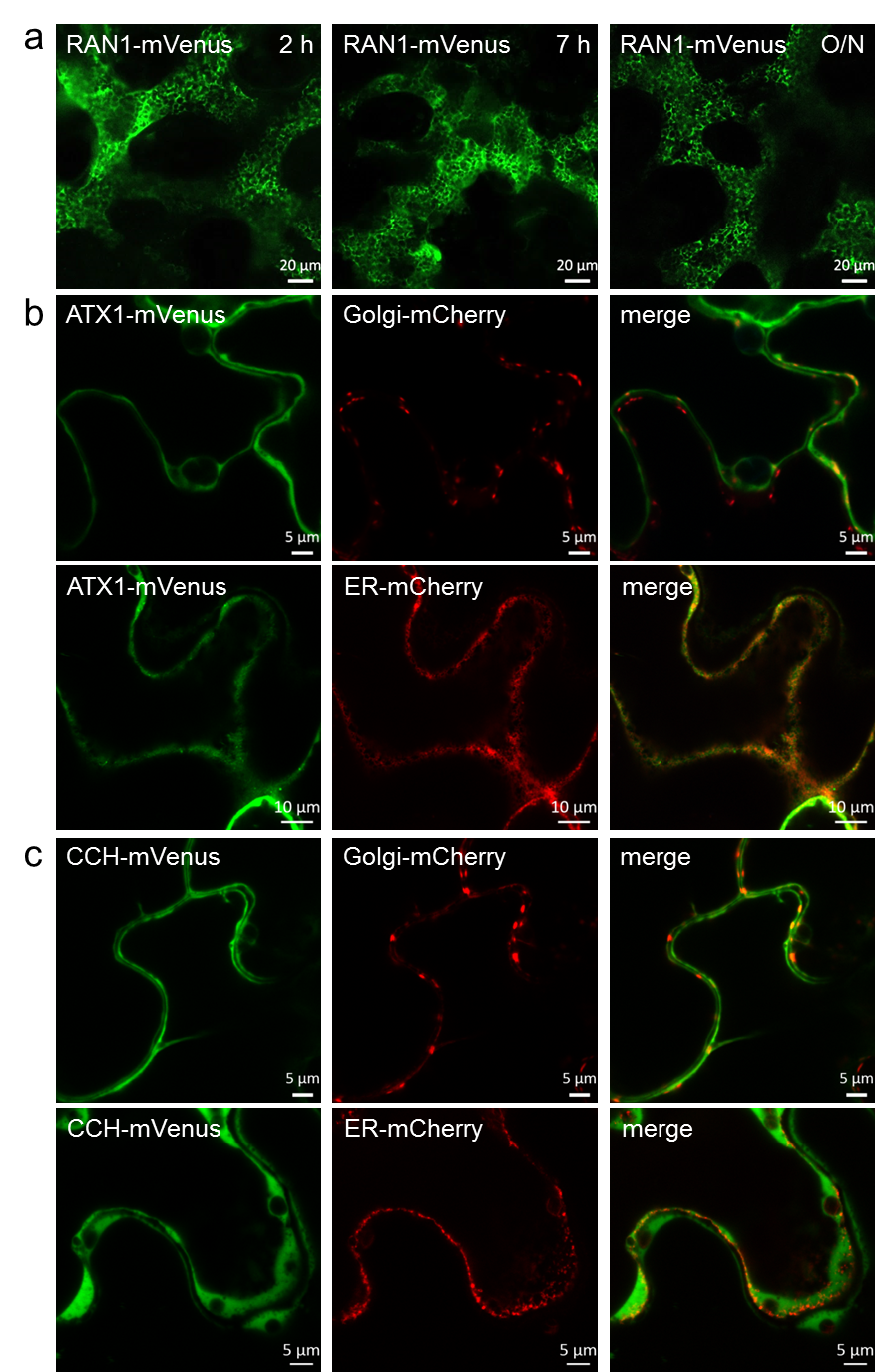


Figure S1: Subcellular localization studies on RAN1, ATX1 and CCH in *N. benthamiana* leaf cells. Related to figure 1. **A)** Coexpression of RAN1-mVenus with an ER-mCherry marker at different time points post induction. **B)** Coexpression of mVenus-tagged copper chaperone ATX1 with Golgi-mCherry and ER-mCherry marker. ATX1 shows no colocalization with any of the marker proteins supporting cytosolic localization of the chaperone characteristic for soluble copper chaperones. **C)** Coexpression of mVenus-tagged copper chaperone CCH with Golgi-mCherry and ER-mCherry marker. Similar to ATX1 the plant specific copper chaperone CCH shows no colocalization with any of the marker proteins supporting cytosolic localization of the chaperone characteristic for soluble copper chaperones.

Figure S2
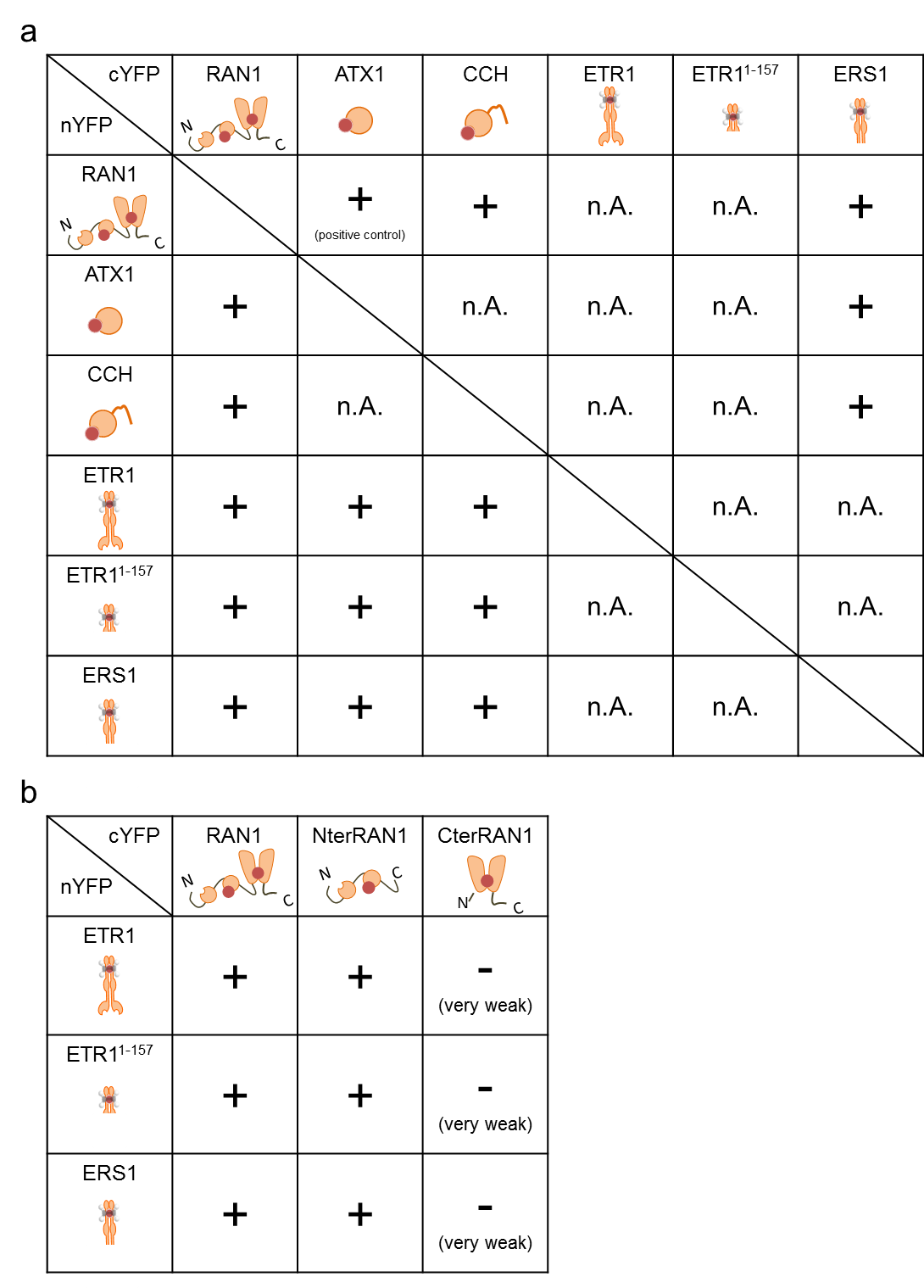


Figure S2: Summary of Bimolecular Fluorescence Complementation (BiFC) studies, revealing interaction of ethylene receptors with both, soluble and membrane bound copper carriers. Related to figures 2 and 3. **A, B)** BiFC studies on the interaction of RAN1, ATX1-like copper chaperone and ethylene receptors. Proteins where fused either with fragment nYFP or cYFP as indicated. + indicates fluorescence complementation, − indicates very weak complementation most likely resulting from spontaneously assembled YFP as a result of stochastic proximity. n.a. construct not tested. The RAN1-ATX1 interaction represents a positive control as it described in ^16^.

Figure S3


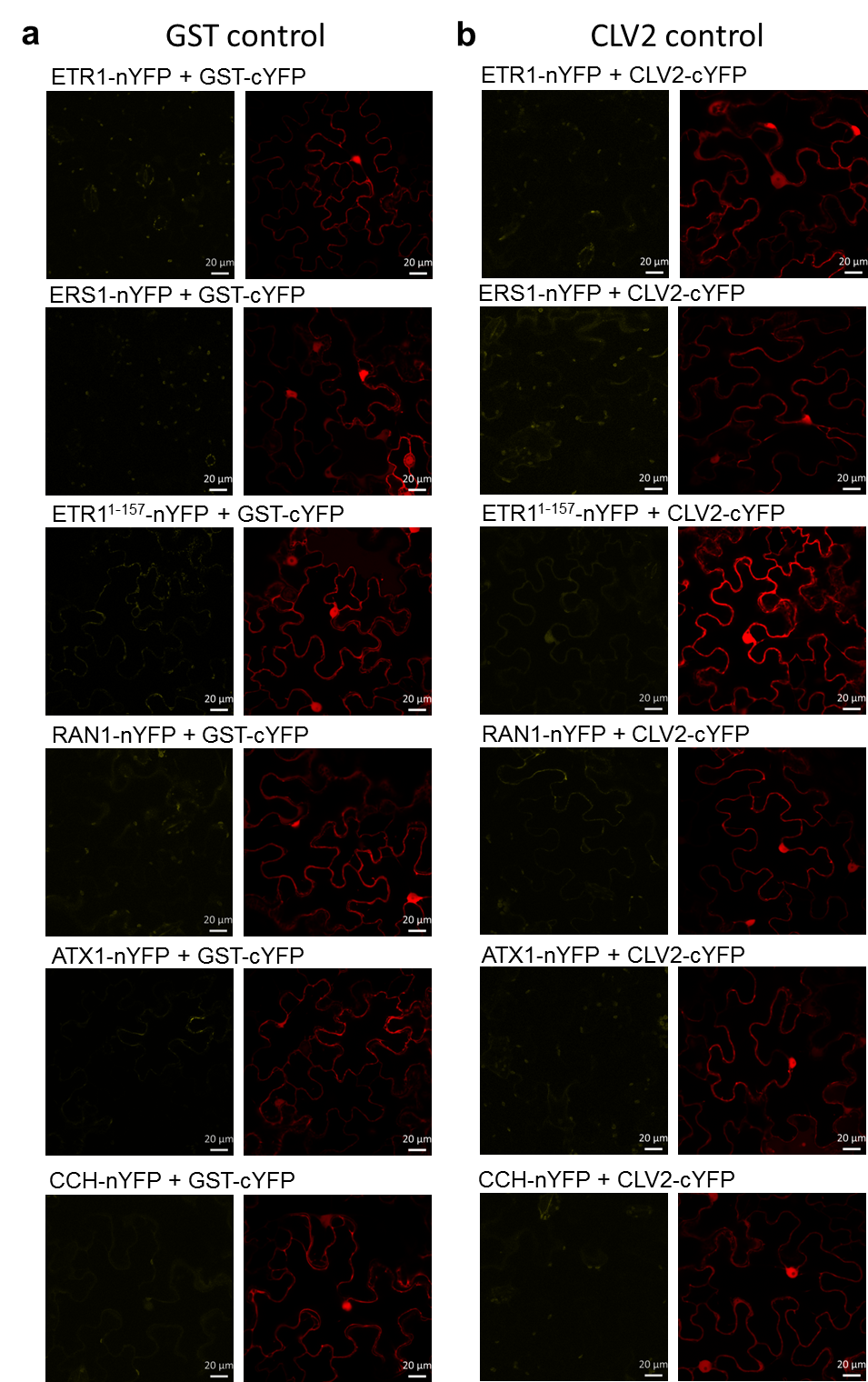


Figure S3 BiFC negative controls. Related to figures 2 and 3. nYFP-fusions of all proteins used in this study with cYFP-fusion of either cytosolic glutathione-S-transferase (GST, left panel) or the ER localized **receptor-like protein CLAVATA2 (CLV2, right panel) were used in bimolecular fluorescence complementation assay.** No YFP fluorescence emission was detected in any of the controls.

Figure S4


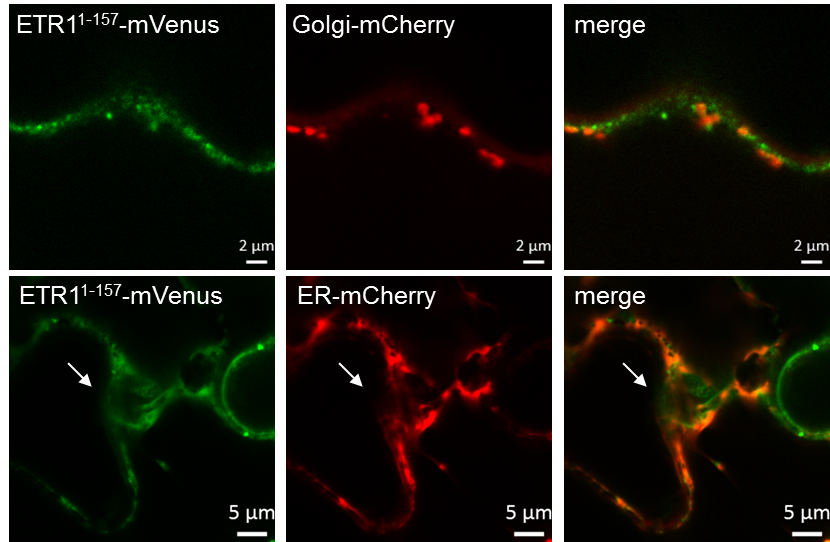


Figure S4: Subcellular localization of ETR1^1-157^ in *N. benthamiana* cells expressing ETR1-mVenus and Golgi-mCherry (upper pannel) or ER-mCherry (lower pannel). ETR1^1-157^ colocalizes partially with the ER-marker, but no clear colocalization with the Golgi-marker could be observed. Related to figures 2 and 3. In addition to colocalization with the ER marker ETR1^1‑157^ localizes also to other membranous structures as indicated by the arrow.

Figure S5


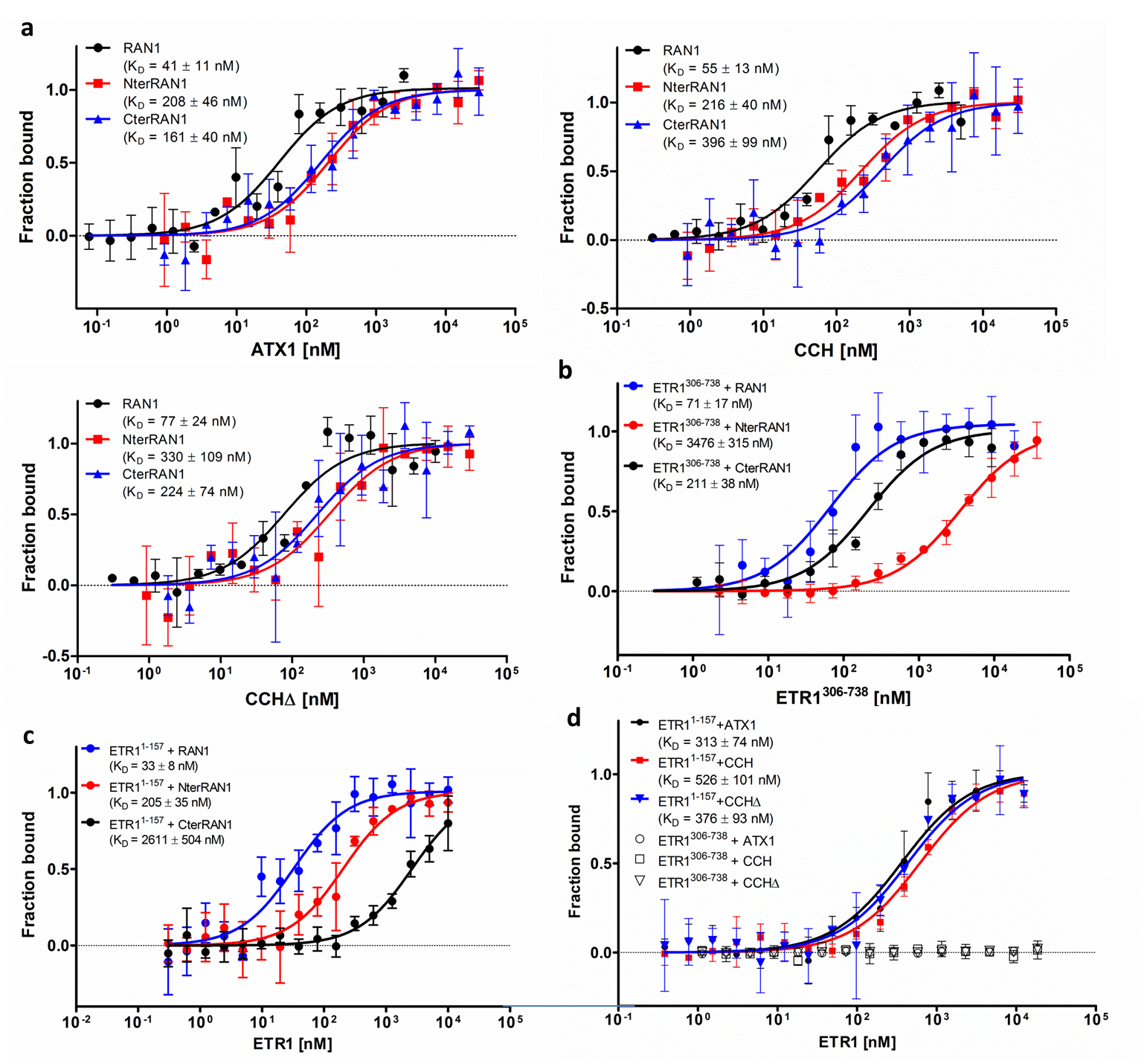


Figure S5: Quantitative binding studies of RAN1 and ATX1-like copper chaperones by microscale thermophoresis*.* Related to figure 4. **A)** Binding studies between RAN1, the N-terminal domain of RAN1 (NterRAN1) and RAN1 lacking the N-terminal domain (CterRAN1) with soluble copper chaperones ATX1, CCH and CCH lacking the C-terminal extension (CCHΔ). The dissociation constants (K_D_) determined in these studies indicate tight interaction between RAN1 and soluble copper chaperones (K_D_ = 41 – 77 nM). RAN1 truncation mutants show an approx. 4-times lower affinity, although still in the nM range (NterRAN: K_D_ = 208 – 330 nM, CterRAN1: K_D_ = 161 – 396 nM). Notably, deletion of the C-terminal extension of CCH results only in a slight decrease in affinity. This data suggest that soluble chaperones interact with the N-terminal ATX1-like domains as well as with the copper translocating region of RAN1. **B)** Binding studies with ETR1^306-738^ demonstrate interaction with RAN1 (K_D_ = 71 ± 17 nM), NterRAN1 (K_D_ = 3476 ± 315 nM) and CterRAN1 (K_D_ = 211 ± 38 nM) indicating additional binding sites for the ETR1-RAN1 interaction between the extra-membranous part of the receptor and the C-terminal part of RAN1. **C)** Titration of ETR1^1‑157^ with RAN1, NterRAN1 and CterRAN1 demonstrates the importance of the N-terminal ATX1-like motifs for tight interaction of ETR1 and RAN1. Full-length and NterRAN1 show affinities for ETR1^1-157^ in the nM range (RAN1: 33 ± 8 nM, NterRAN1 205 ± 35 nM). In contrast, dissociation constant K_D_ drops to the µM range with CterRAN1 (2611 ± 504 nM). **D)** Titration of ETR1 with soluble copper chaperones demonstrates direct interaction of receptors and soluble chaperones in the upper nM-range (ATX1: 313 nM, CCHΔ: 376 nM). Data indicate that the C-terminal extension of CCH partially shields or destabilizes this interaction (K_D_ = 526 nM). In contrast to binding studies with RAN1, ETR1^306-738^ shows no binding to ATX1, CCH or CCHΔ indicating that the interaction of ETR1 with the soluble chaperones of the ATX1 family is determined by the transmembrane region of the receptor.

Figure S6
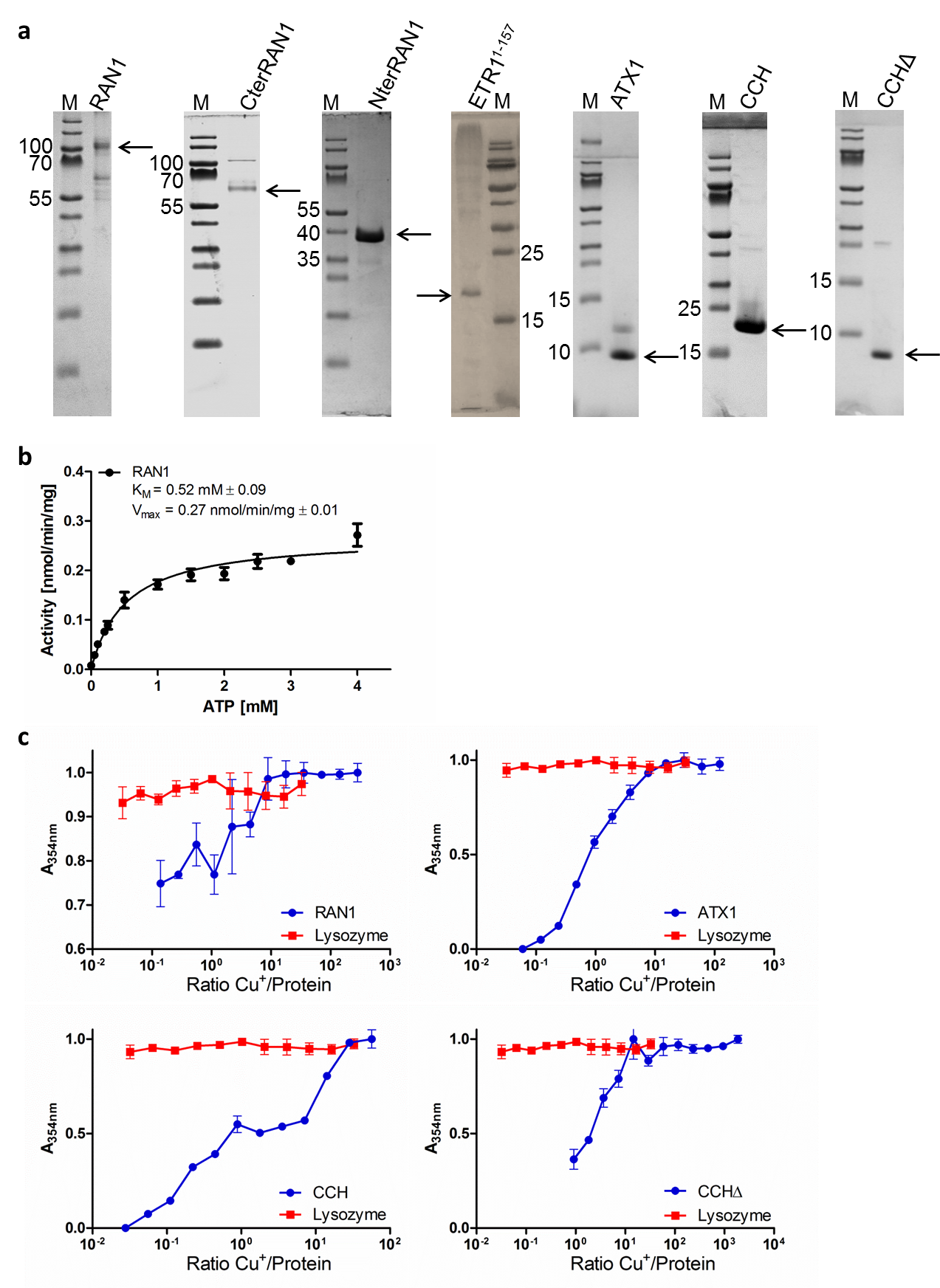


Figure S6: Heterologous expression and purification of RAN1, NterRAN1, CterRAN1, ETR1^1-157^, ATX1, CCH and CCHΔ. Related to figure 4 and Material and Methods: Purification of recombinant protein. **A)** SDS-PAGE of purified recombinant proteins; M: PageRuler Prestained Protein Ladder (Thermo Scientific). 1 µg of purified protein was loaded on Tris-Tricine gels and separated for 2.5 h at 30 mA. Gels were washed three times with hot water, afterwards proteins were stained overnight using colloidal Coomassie G-250. Image acquisition was performed using a BioDocAnalyzer System (Analytik Jena) with standard settings. Each gel slice originates from a different gel. Only lanes unrelated to this publication or empty were cropped. **B)** ATPase assay of RAN1 for quantification of ATP hydrolysis activity. 20 µl of RAN1 was stepwise diluted in 50 mM HEPES, 300 mM NaCl pH 7.5 and hydrolysis initiated by the addition of 5 µl ATP (0 – 4 mM). Reaction was stopped after 25 min by the addition of 175 µl of 1 M H_2_SO_4_. 50 µl of malachite green staining solution (0.122 % malachite green in 20% H_2_SO_4_) were added and incubated under vigorous shaking for 10 min. Absorbance was measured at 620 nm to quantify released phosphate. **C)** Copper binding studies on RAN1, CCH, ATX1 and CCHΔ. Functional folding of purified recombinant proteins was probed by copper binding studies using copper-BCA. Stepwise dilution of 50 µl protein solution in 50 mM HEPES, 300 mM NaCl pH 7.5 was titrated against a defined concentration of 50 µl copper (50 µM copper(I) as Cu-BCA_2_-complex) and copper transfer from Cu-BCA_2_-complex to the protein was monitored by measuring the absorbance at 354 nm. This assay was used as a qualitative method only to demonstrate copper(I) binding ability of RAN1, CCH, ATX1 and CCHΔ.
